# Gd-IgA1-targeting therapy in IgA nephropathy ~what is pathogenic Gd-IgA1?~

**DOI:** 10.3389/fneph.2026.1977707

**Published:** 2026-10-01

**Authors:** Kazuaki Mori, Yoshihito Nihei, Yusuke Suzuki

**Affiliations:** Department of Nephrology, Juntendo University Faculty of Medicine, Tokyo, Japan

**Keywords:** B cells, B-cell-targeted therapies, Gd-IgA1, IgA nephropathy, mucosal immunity

## Abstract

IgA nephropathy (IgAN) is the most common form of primary glomerulonephritis worldwide, and galactose-deficient IgA1 (Gd-IgA1) is widely recognized as the central pathogenic molecule in the current multi-hit model of IgAN. Over the past few decades, advances in our understanding of IgAN pathogenesis have driven the development of mechanism-based therapies targeting Gd-IgA1-producing cells and the pathways that regulate their differentiation and survival. Recent clinical trials have demonstrated marked reductions in circulating Gd-IgA1, accompanied by significant improvements in proteinuria and preservation of kidney function, highlighting the transition from non-specific disease-modifying therapies toward mechanism-based therapies in IgAN. However, optimizing the use of these emerging therapies requires a deeper understanding of the immune mechanisms responsible for pathogenic Gd-IgA1 production. In this review, we summarize current knowledge regarding the cellular sources and molecular pathways responsible for the production of Gd-IgA1, review recent advances in therapies targeting these cells, and discuss the emerging concept that specific Gd-IgA1 fractions, including IgA-type anti-mesangial cell autoantibodies (IgA-MESCA), may contribute to the disease pathogenesis.

## 1 Introduction

IgA nephropathy (IgAN) is a mesangioproliferative glomerulonephritis characterized by dominant mesangial IgA deposition and is the most common primary glomerulonephritis worldwide (1–3).

Recent data have shown that most patients progress to kidney failure within 10–15 years after disease onset (3, 4), and IgAN remains one of the leading causes of end-stage kidney failure (2, 3).

Early studies demonstrated that IgAN patients have increased circulating levels of galactose-deficient IgA1 (Gd-IgA1) (5, 6) (first hit). Gd-IgA1 subsequently forms high-molecular-weight immune complexes (ICs) with IgG and/or IgA antibodies directed against Gd-IgA1 (7) (second and third hits), and these ICs are then deposited in the mesangial region (8) (fourth hit). This multi-hit hypothesis (9) is now widely accepted, and Gd-IgA1 production is considered a central pathogenic mechanism in IgAN (10, 11). Accordingly, therapeutic strategies targeting Gd-IgA1 are currently under active development.

In this review, we first discuss the cell types responsible for Gd-IgA1 production and the mechanisms underlying their dysregulation. We then review current and emerging therapies targeting these cells for IgAN. Finally, we highlight recent advances in our understanding of pathogenic Gd-IgA1.

## 2 Identification of Gd-IgA1 in IgAN

IgAN patients are known to exhibit significantly higher circulating IgA levels than non-IgAN individuals (12). However, IgA deposition within the mesangial region is not specific to IgAN and can also be observed in other conditions including lupus nephritis, hepatitis B virus–associated nephropathy, and liver cirrhosis (13–15). Therefore, elucidation of IgA abnormalities specific to IgAN has become an important research focus.

In 1997, Tomana and colleagues analyzed O-glycan structures of serum IgA1 by gas chromatography and identified IgA1 with galactose-deficient O-glycans in the hinge region (5), which was subsequently termed Gd-IgA1. They further showed that Gd-IgA1 could be detected using N-acetylgalactosamine (GalNAc)-specific lectin Helix aspersa agglutinin (HAA), as the galactose deficiency in the O-linked glycans of the IgA1 hinge region results in exposure of GalNAc (5). Hiki and colleagues further analyzed the O-glycan structure of IgA1 deposited in the glomeruli of IgAN patients using mass spectrometry and revealed that the deposited IgA1 is also characterized by reduced galactosylation and sialylation (11).

We further generated monoclonal antibody KM55, which specifically recognizes the hinge region of human Gd-IgA1, and directly demonstrated that glomerular IgA deposits in IgAN consist predominantly of Gd-IgA1 (8).

## 3 Mucosal immune dysregulation in Gd-IgA1 production

The mucosal immune system has been considered a predominant site where B cells producing pathogenic IgA are primed in IgAN patients (16–18). Evidence supporting the involvement of mucosal immunity comes from both experimental and clinical observations. Experimentally, numerous studies have demonstrated that IgAN can be induced by oral antigens (19–22). Glomerular IgA deposits contain J chain, suggesting that the deposited IgA is present in a polymeric form (23–25), and secretory component has also been detected in a subset of glomerular IgA deposits, indicating their potential mucosal origin (25, 26). Clinically, many patients experience episodic gross hematuria after mucosal infections such as upper respiratory or gastrointestinal infections (13, 27), supporting the involvement of mucosal immune responses in disease activity. Consistent with this concept, therapeutic interventions targeting mucosal immunity, including tonsillectomy (2, 3, 28, 29) and Nefecon (30–32), have been shown to improve renal outcomes. A genome-wide association study (GWAS) has further identified multiple susceptibility loci related to mucosal immunity in patients with primary IgAN, including CARD9 and HORMAD2, which are also associated with inflammatory bowel disease (33).

These findings suggest that dysregulation of mucosa-associated lymphoid tissue (MALT), particularly nasopharynx-associated lymphoid tissue (NALT) and gut-associated lymphoid tissue (GALT), plays an important role in Gd-IgA1 production in IgAN. Within NALT, upper airway mucosal lymphoid tissues centered around the tonsils are considered important sites, whereas Peyer’s patches in the terminal ileum are regarded as key sites within GALT (13, 17). The predominant MALT involved in IgAN may vary across geographical regions.

## 4 B-cell activation pathways regulating Gd-IgA1 production

Within mucosal lymphoid tissues, B-cell class switch recombination (CSR) and differentiation into IgA-producing cells are regulated by germinal center (GC)-dependent responses and signaling pathways involving toll-like receptor (TLRs), a proliferation-inducing ligand (APRIL), and B-cell activating factor (BAFF) (34, 35). A subset of these IgA-producing cells is thought to produce Gd-IgA1 in IgAN.

### 4.1 Germinal center-dependent B-cell responses in Gd-IgA1 production

Exogenous antigens are taken up by antigen-presenting cells (APCs), primarily dendritic cells (DCs), and activate naïve CD4^+^ T cells through major histocompatibility complex (MHC) class II-dependent antigen presentation (36). Subsequently, a subset of activated T cells differentiates into follicular helper T (Tfh) cells (36, 37), which provide helper signals to B cells within GCs (38–40).

Tfh cell-derived CD40 ligand (41–43) and transforming growth factor-β (34, 42, 44) play central roles in CSR to IgA, and interleukin-21 (IL-21) (38, 45) and interleukin-10 (34, 42) further support the expansion, survival, and differentiation of the IgA-switched B cells into antibody-secreting cells (ASCs). During the GC reaction, B cells undergo somatic hypermutation, followed by antigen-driven selection that results in affinity maturation and the generation of high-affinity B-cell receptors. Subsequently, some of these cells persist as memory B cells, whereas others differentiate into long-lived plasma cells (LLPCs) that migrate to survival niches, where they are maintained through survival signals, including APRIL–B-cell maturation antigen (BCMA) signaling (46, 47).

In IgAN patients, several studies have shown that increased frequencies of activated Tfh cells and elevated serum IL-21 levels are associated with higher serum Gd-IgA1 concentrations (48, 49). In addition, IL-21 produced by circulating Tfh cells has been reported to correlate with disease activity, including the degree of proteinuria (48, 50, 51).

These findings support a potential role for GC responses in Gd-IgA1 production, although definitive evidence that GC reactions represent the predominant source of pathogenic Gd-IgA1-producing cells is still lacking.

### 4.2 Involvement of toll-like receptor signaling in Gd-IgA1 production

TLRs, major pattern-recognition receptors broadly expressed by innate immune cells, including APCs, have attracted considerable attention as they not only recognize pathogen-derived molecular patterns to induce inflammatory cytokine production and immune responses but also regulate B-cell responses (52).

Among these receptors, TLR9, TLR7, and TLR4 have been implicated in the pathogenesis of IgAN.

TLR9 recognizes unmethylated CpG DNA derived from microorganisms. We previously demonstrated that TLR9 stimulation promotes Gd-IgA1 production through induction of interleukin-6 (IL-6) and APRIL and exacerbates glomerular IgA, IgG, and C3 deposition as well as kidney injury in gddY mice, a spontaneous murine model of IgAN (53). Furthermore, TLR9 polymorphisms and aberrant TLR9 expression have been associated with disease severity and therapeutic responsiveness in IgAN (54), and tonsillar TLR9 expression has been linked to the efficacy of steroid therapy combined with tonsillectomy (55).

Similar to TLR9, activation of TLR7, which recognizes microbial single-stranded RNA, has also been shown to aggravate IgAN by promoting Gd-IgA1 production (56, 57). TLR7 expression is associated with IL-6 production and correlates with APRIL expression in the tonsils of IgAN patients, suggesting an important role for TLR7 in the disease pathogenesis (56, 57).

Consistent with these concepts, hydroxychloroquine has been reported to produce a sustained reduction in proteinuria in IgAN patients, and suppression of TLR7/9 signaling has been suggested as one of the mechanisms underlying its therapeutic effects (57–59).

TLR4, which recognizes lipopolysaccharide from Gram-negative bacteria, has been proposed to contribute to IgAN pathogenesis through activation of the TLR4/MyD88/NF-κB signaling pathway in response to gut dysbiosis (60–62). Although the therapeutic effects of TLR4 inhibition have not yet been sufficiently investigated in IgAN, these findings suggest that modulation of TLR4 signaling could also be a possible therapeutic strategy.

### 4.3 APRIL/BAFF-mediated regulation of B-cell differentiation and Gd-IgA1 production

APRIL and BAFF, whose expression can be enhanced by TLR signaling in IgAN, play pivotal roles in the differentiation of B cells into IgA-producing cells (13, 34). These cytokines are produced by a variety of cell types, including monocytes, DCs, and epithelial cells (34, 63, 64), and regulate B-cell survival, maturation, and differentiation, while also promoting CSR to IgA and the maintenance of ASCs (34, 65, 66).

Three receptors mediate APRIL/BAFF signaling: BAFF receptor (BAFFR), which specifically binds BAFF, and the two shared receptors, transmembrane activator and calcium-modulator and cyclophilin ligand interactor (TACI) and BCMA (64, 66). Among these receptors, BCMA exhibits a higher affinity for APRIL than for BAFF, whereas TACI binds APRIL and BAFF with comparable affinity (66). Immature and naïve B cells express high levels of BAFFR, and BAFF–BAFFR signaling is critical for their survival and maintenance (64, 67). In contrast, BAFFR expression declines as B cells differentiate into plasmablasts and plasma cells, while APRIL signaling through TACI and particularly BCMA becomes increasingly important (64, 67). Consequently, BAFF is thought to play a predominant role in the survival and maintenance of immature and mature B cells, whereas APRIL is more closely involved in IgA CSR and the maintenance of ASCs.

Experimental evidence also supports the involvement of the APRIL/BAFF pathway in IgAN. We previously demonstrated that TLR9 and TLR7 activation promoted APRIL-dependent Gd-IgA1 production in IgAN-prone gddY mice (53, 56, 57). TLR4 activation has also been suggested to upregulate APRIL and BAFF expression in murine models of IgAN (61, 62). The pathogenic significance of this pathway is further supported by GWAS, which have identified APRIL- and TACI-related loci as susceptibility loci for IgAN (68–70). Moreover, recent clinical trials showing that APRIL/BAFF-targeted therapies markedly reduce serum Gd-IgA1 levels and proteinuria further support the role of this pathway in sustaining pathogenic Gd-IgA1 production in IgAN (71–75).

## 5 B-cell types responsible for Gd-IgA1 production

As described above, mucosal immune dysregulation promotes the differentiation of activated B cells into Gd-IgA1-producing cells. Once mucosal immune dysregulation is initiated, IgAN typically follows a chronic course without spontaneous remission. This clinical course suggests that persistent production of Gd-IgA1 cannot be explained solely by local mucosal immune responses. Therefore, identifying where Gd-IgA1-producing cells reside and which immune compartments sustain Gd-IgA1 production is essential for understanding the pathogenesis of IgAN.

IgAN patients exhibit increased numbers of polymeric IgA1-positive plasma cells, IgA1-producing cells, and J chain mRNA–expressing IgA-producing B cells in the bone marrow (27, 76). Moreover, experimental studies have shown amelioration of IgAN following bone marrow transplantation in murine models (77, 78), and several clinical cases have reported remission after bone marrow or peripheral blood stem cell transplantation for hematologic disorders (79–81). Furthermore, it has been demonstrated that antigen-specific lymphocytes and APCs undergo continuous trafficking between mucosal tissues and bone marrow in humans (13). Taken together, dysregulation of the mucosa–bone marrow axis has been proposed to promote the mis-homing of Gd-IgA1-producing cells aberrantly primed in MALT to the bone marrow, where they differentiate into LLPCs and continuously produce Gd-IgA1 (27).

Recent studies have also identified infiltration of B cells and plasma cells within renal tissues of IgAN patients (51, 56, 82). These intrarenal B cells have been reported to express TLR7 and produce IL-6 (56), both of which promote Gd-IgA1 production as described above (53, 56, 57), raising the possibility that these cells might contribute to local Gd-IgA1 production and the pathogenesis of IgAN. In addition, increased circulating gut-homing B cells expressing IgA have been reported in IgAN patients (83). More specifically, B cells expressing Gd-IgA1 have been identified in the peripheral blood of IgAN patients, with a predominance of λ light chains consistent with mesangial IgA deposits (84). These cells express mucosal homing receptors, suggesting their potential to home to mucosal tissues (84). Further studies, including investigation of other potential sources of Gd-IgA1 such as breast milk (85), are warranted to demonstrate these possibilities.

The mechanisms underlying the production and long-term maintenance of Gd-IgA1-producing cells are summarized in **Figure 1**.

**Figure 1:**
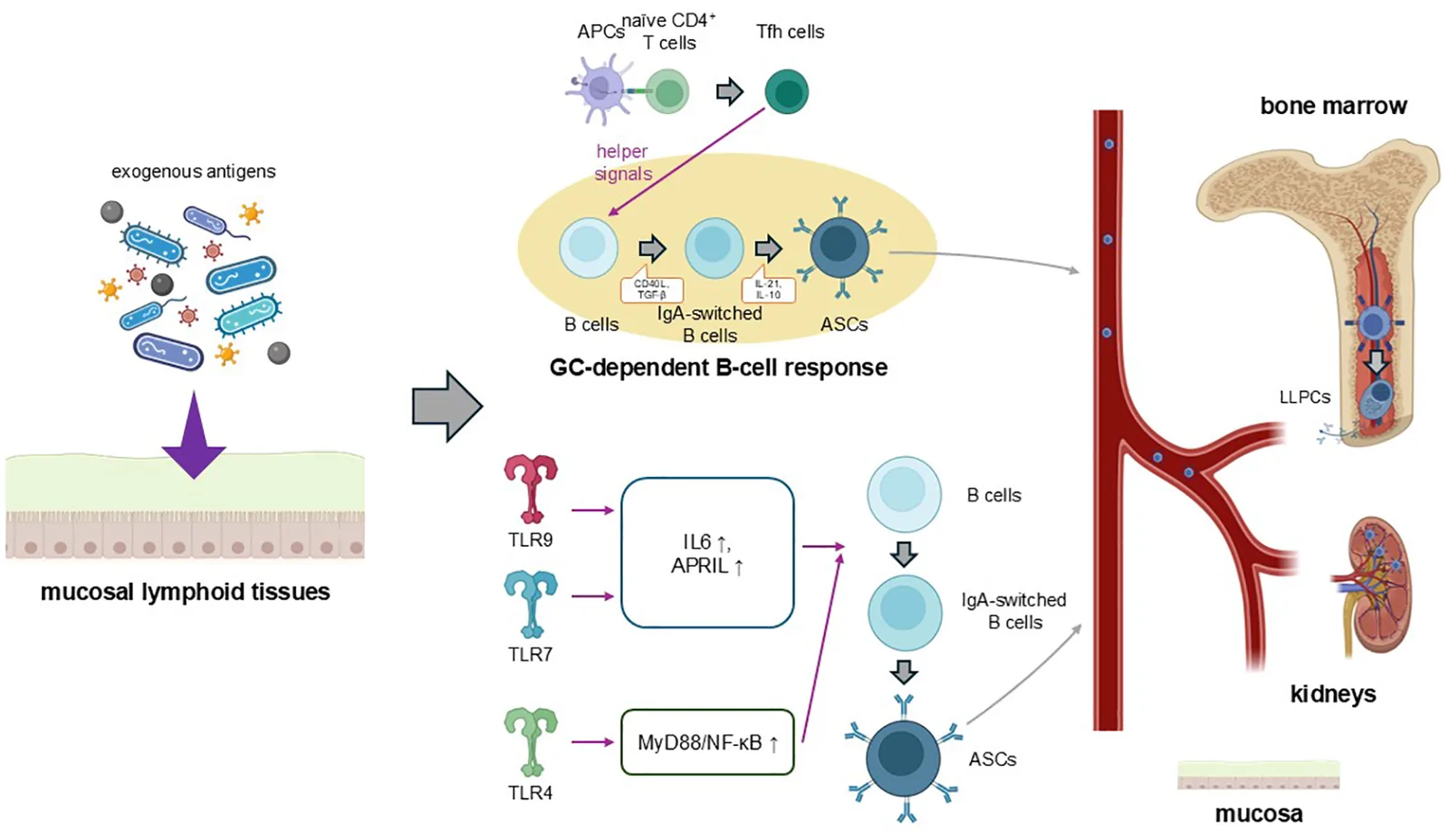
Current understanding of the mechanisms underlying the generation and long-term maintenance of Gd-IgA1-producing cells in IgA nephropathy. Exogenous antigen exposure in mucosal lymphoid tissues promotes the generation of IgA-switched B cells through GC-dependent and TLR-mediated B-cell responses. A subset of these cells differentiates into Gd-IgA1-producing ASCs, which may subsequently migrate to the bone marrow, where they differentiate into LLPCs and sustain Gd-IgA1 production. Gd-IgA1-producing ASCs might also home to the kidney and mucosal tissues, potentially contributing to Gd-IgA1 production. APCs, antigen-presenting cells; Tfh, follicular helper T; CD40L, CD40 ligand; TGF-β, transforming growth factor-β; IL, interleukin; Gd-IgA1, galactose-deficient IgA1; ASCs, antibody-secreting cells; GC, germinal center; TLR, toll-like receptor; APRIL, a proliferation-inducing ligand; MyD88, myeloid differentiation primary response 88; NF-κB, nuclear factor kappa B; LLPCs, long-lived plasma cells.

## 6 Therapeutic strategies

Advances in the understanding of IgAN pathogenesis have fundamentally reshaped therapeutic strategies for the disease. Whereas treatment has traditionally relied largely on non-specific immunosuppression and supportive care, emerging therapeutic approaches increasingly target pathogenic Gd-IgA1-producing cells. Based on the pathogenic framework described above, these strategies can be broadly considered from two complementary perspectives: targeting the mucosal immune abnormalities that initiate pathogenic Gd-IgA1 production and targeting specific stages of B-cell differentiation and survival that sustain it.

### 6.1 Therapeutic strategies targeting mucosal immunity

Tonsillectomy has been performed with the aim of reducing mucosa-derived Gd-IgA1 production and suppressing the systemic dissemination of the aberrant ASCs. Both steroid therapy and tonsillectomy have been shown to independently contribute to the achievement of clinical remission (29), and this combined therapeutic approach subsequently became widely adopted across medical institutions in Japan (86). Tonsillectomy alone reduced serum Gd-IgA1 levels and improved hematuria in 59% of patients (87). However, in some patients, reductions in serum Gd-IgA1 levels were observed with additional steroid therapy after tonsillectomy (87). Furthermore, steroid therapy combined with tonsillectomy demonstrated higher rates of proteinuria remission (65.7% vs. 35.0%) and hematuria remission (77.1% vs. 45.0%) compared with steroid therapy alone, and achieved a higher overall urinary remission rate (54.3% vs. 25.0%) (88). These findings indicate that effective control of IgAN may require a combination of targeting the disease-associated mucosal tissue and systemically regulating the Gd-IgA1-producing cells. Currently, both Japanese guidelines and the KDIGO 2025 Guideline indicate that tonsillectomy may be considered in patients expected to benefit from improvement in urinary findings and potentially renal prognosis, particularly in those showing worsening urinary abnormalities such as hematuria following upper respiratory tract infections (2, 3). The KDIGO 2025 Guideline also recommends considering tonsillectomy as a treatment option only for Japanese patients (3).

Nefecon is a targeted-release formulation of budesonide designed to deliver the drug to the distal ileum, where sustained local release of the active compound occurs (30). Budesonide possesses approximately fivefold greater anti-inflammatory potency than prednisolone; however, owing to its extensive first-pass hepatic metabolism, systemic bioavailability remains approximately 10% (89). Accordingly, Nefecon is considered to selectively modulate abnormal intestinal mucosal immunity centered on GALT while minimizing systemic adverse effects. Treatment with Nefecon significantly reduced serum Gd-IgA1 by approximately 18% and IgA–IgG IC by approximately 7% (90), decreased proteinuria by approximately 27% at 9 months, and attenuated the decline in eGFR (30), supporting direct intervention against pathogenic Gd-IgA1 production. Subsequent large-scale clinical trials confirmed an approximately 27% reduction in proteinuria together with preservation of kidney function, and demonstrated sustained renoprotective effects over 2 years, with a between-group difference in eGFR of 5.05 mL/min/1.73 m^2^ (31, 32). However, it should be noted that Nefecon discontinuation results in re-elevation of serum Gd-IgA1 levels (91), accompanied by recurrence of proteinuria and a decline in eGFR (3), and that its long-term efficacy remains to be established. Following accelerated approval by the U.S. FDA in 2021, Nefecon received full approval in December 2023 and has subsequently been approved in Europe (92) and several Asian countries.

### 6.2 Therapeutic strategies targeting different stages of B-cell differentiation

The efficacy of B-cell–targeted therapies depends on which stage of B-cell differentiation is responsible for Gd-IgA1 production. In a randomized clinical trial of rituximab in IgAN patients, peripheral CD20-positive B cells were effectively depleted; however, no significant improvements were observed in serum Gd-IgA1 levels, proteinuria, or kidney function after 1 year of treatment (93). This finding suggests that CD20-negative Gd-IgA1-producing cells, such as differentiated plasma cells, and the pathways that regulate their differentiation and survival may play a pivotal role in the disease pathogenesis.

B-cell–targeted therapies can be broadly classified into two categories: B cell/ASC–depleting therapies, which directly eliminate B cells or ASCs, and B cell–modulating therapies, which regulate B-cell function (94, 95). The former include CD38-targeted therapies, and the latter include APRIL/BAFF-targeted and Bruton’s tyrosine kinase (BTK)-targeted therapies.

Atacicept, a TACI-Fc fusion protein that inhibits both APRIL and BAFF, reduced serum Gd-IgA1 levels by approximately 60% at week 24 in IgAN patients, and this reduction was sustained through week 72 (71). An approximately 50% reduction in proteinuria at week 72 and preservation of kidney function were also observed, and reductions in serum Gd-IgA1 levels were correlated with improvements in proteinuria (71, 72). Povetacicept, another dual APRIL/BAFF inhibitor, also demonstrated reductions in serum Gd-IgA1 levels (57% reduction at week 12 and 77% reduction at week 48), accompanied by sustained improvement in proteinuria (64% reduction at week 48), improvement or resolution of hematuria (approximately 90%), preservation of kidney function, and clinical remission (53%) (73).

Sibeprenlimab, an anti-APRIL monoclonal antibody, reduced serum Gd-IgA1 levels by approximately 65% at 3 months in a clinical trial and achieved up to a 62.0% reduction in proteinuria at 12 months while also demonstrating stabilization of kidney function (74). Similarly, zigakibart, another APRIL-targeting monoclonal antibody, produced a 74% reduction in serum Gd-IgA1 levels, together with a 60% reduction in proteinuria at 100 weeks and sustained stabilization of eGFR (75).

These findings suggest that selective inhibition of APRIL alone may also provide effective disease control in IgAN. In contrast, belimumab, an anti-BAFF monoclonal antibody, has not demonstrated clear clinical efficacy in IgAN patients, and no further clinical development for IgAN has been reported. These results indicate that APRIL, rather than BAFF, may play a central role in the pathogenesis of IgAN.

Felzartamab, an anti-CD38 monoclonal antibody that aims to eliminate pathogenic ASCs, reduced serum Gd-IgA1 levels and proteinuria by up to 44.8% at 9 months, with the effect maintained at 24 months (44.5% reduction), and attenuated eGFR decline (96). Likewise, mezagitamab, another anti-CD38 monoclonal antibody, is currently under clinical investigation for IgAN, and reductions in Gd-IgA1-related biomarkers and proteinuria have been reported (97).

In addition, rilzabrutinib, an inhibitor targeting BTK, a key intracellular signaling molecule required for B-cell activation (98, 99), is currently being evaluated in clinical trials for IgAN and may provide a novel B-cell–targeted therapeutic approach.

Overall, targeting the cellular types within the B-cell lineage responsible for pathogenic Gd-IgA1 production may enable more effective disease control, thereby offering the potential for curative therapy during the reversible, immunologically active stage of IgAN (100).

The therapeutic strategies described above are summarized in **Table 1**.

**Table 1:** Emerging therapeutic strategies targeting mucosal immunity and B-cell differentiation.

Therapeutic targets		Treatment	Gd-IgA1	UPCR	eGFR
mucosal immunity	GALT	Nefecon	↓18% vs ↑10% with placebo (90)	↓40.3% vs ↑1.0% with placebo (32)	↓2.47 vs ↓7.52 mL/min/1.73 m^2^ with placebo (32)
B-cell differentiation	APRIL/BAFF	atacicept	↓60% vs ↑2% with placebo (71)	↓50% vs ↑25% with placebo (71)	↑12% vs ↓25% with placebo (71)
		povetacicept	↓77% (73)	↓64% (73)	↑3.2 mL/min/1.73 m^2^ (73)
	APRIL	sibeprenlimab	↓65% vs ↑20% with placebo (74)	↓62% vs ↓20% with placebo (74)	↑0.2 vs ↓7.4 mL/min/1.73 m^2^ with placebo (74)
		zigakibart	↓74% (75)	↓60.4% (75)	↑0.48 mL/min/1.73 m^2^/year (75)
	CD38	felzartamab	↓43.2% vs ↓6.8% with placebo (96)	↓44.5% vs ↑3.7% with placebo (96)	↓6.4 vs ↓15.2 mL/min/1.73 m^2^ with placebo (96)

GALT, gut-associated lymphoid tissue; APRIL, a proliferation-inducing ligand; BAFF, B-cell activating factor; Gd-IgA1, galactose-deficient IgA1; UPCR, urine protein-to-creatinine ratio.

## 7 Unresolved questions in the pathogenicity of Gd-IgA1

The preceding sections have highlighted the disease-specific characteristics of Gd-IgA1. Nevertheless, several important questions remain unresolved.

Some relatives of patients with familial IgAN have been reported to have increased serum Gd-IgA1 levels without developing IgAN (101). In mice with B cell-specific *c1galt1* deletion, markedly elevated serum Gd-IgA1 did not increase glomerular IgA deposition, whereas mucosa-derived IgA1 from IgAN patients showed greater mesangial deposition irrespective of its Gd-IgA1 content (102). No significant difference in long-term kidney outcomes was observed despite greater reductions in serum Gd-IgA1 with higher-dose steroid therapy (103), and an experimental study of anti-APRIL therapy further revealed that, although proteinuria decreased in parallel with improvement in IgA glycosylation during treatment, this reduction was maintained even after abnormal IgA glycosylation re-emerged following treatment discontinuation (104). Taken together, these findings indicate that not all Gd-IgA1 is pathogenic. It also remains unclear why Gd-IgA1 selectively deposits in the mesangial region and contributes to the development of IgAN.

The CD89–CD71–TGase2 axis has been proposed to promote mesangial IgA deposition through the interaction of soluble CD89 (sCD89) with transferrin receptor (CD71), an IgA1 receptor on mesangial cells (MCs), with transglutaminase 2 (TGase2) further amplifying this process (105–107). However, because CD71 is expressed not only on MCs but also on a variety of other cell types (108–110), its expression alone is insufficient to explain the selective mesangial IgA deposition, raising the possibility that qualitative properties of Gd-IgA1 may also contribute to its preferential interaction with MCs.

The mesangial IgA-dominant immunoglobulin deposition, a characteristic feature of IgAN, has prompted investigations into the presence of anti-mesangial cell autoantibodies (MESCA). O’Donoghue and colleagues identified IgG autoantibodies against glomerular antigens in IgAN patients and subsequently demonstrated that their target antigens were of mesangial cell origin, establishing the concept of IgG-type MESCA (111, 112). Recently, we discovered IgA-type MESCA in gddY mice and IgAN patients, and identified β2-spectrin (113) and CBX3 (19) as their target autoantigens. β2-Spectrin and CBX3 are widely expressed intracellular proteins, with β2-spectrin predominantly localized to the cytoplasm and CBX3 to the nucleus; however, their aberrant cell-surface expression appears to be restricted to MCs (19, 113, 114). These autoantibodies are also detected with high specificity in IgAN patients and are found to be positive in approximately half of affected individuals (114). Furthermore, they possess the characteristics of Gd-IgA1 and are shown to bind to the autoantigens presented on the MC surface (114). These findings may provide a potential explanation for why only a subset of circulating Gd-IgA1 selectively deposits in the mesangial region. In addition, CBX3-recognizing IgA autoantibodies were shown to exhibit cross-reactivity with specific oral bacterial antigens, suggesting that they may be induced through molecular mimicry following mucosal infections (19).

Overall, the qualitative properties of specific Gd-IgA1 fractions and their interactions with MCs may determine the pathogenicity of Gd-IgA1. Identification of these pathogenic fractions may provide useful biomarkers, particularly given the challenges in developing assays for anti-Gd-IgA1 autoantibodies due to the heterogeneity of O-glycosylation patterns in Gd-IgA1, which makes it difficult to establish a standardized antigen that adequately represents the relevant epitopes (115). The mechanisms by which these pathogenic Gd-IgA1 fractions are generated and maintained, as well as their relative contributions to IgAN pathogenesis, remain to be elucidated.

## 8 Discussion

Advances in our understanding of mucosal immune dysregulation and the cell types responsible for Gd-IgA1 production have transformed the therapeutic landscape of IgAN from relatively non-specific therapies toward mechanism-based interventions.

However, biomarkers capable of identifying the patients most likely to benefit from each therapeutic strategy have yet to be established. Recent findings, including the identification of IgA-type MESCA, further suggest that the pathogenicity of Gd-IgA1 may depend on its qualitative characteristics. Identifying the pathogenic Gd-IgA1 fractions and the cell types responsible for their production may therefore facilitate the development of precision therapies for IgAN.
